# An update on adrenal endocrinology: significant discoveries in the last 10 years and where the field is heading in the next decade

**DOI:** 10.1007/s42000-018-0072-y

**Published:** 2018-11-19

**Authors:** Crystal D. C. Kamilaris, Constantine A. Stratakis

**Affiliations:** Section on Endocrinology and Genetics & Inter-Institute Endocrinology Training Program, Eunice Kennedy Shriver National Institute of Child Health & Human Development (NICHD), National Institutes of Health (NIH), NIH-Clinical Research Center, 10 Center Drive, Building 10, Room 1-3330, MSC1103, Bethesda, MD 20892 USA

**Keywords:** Adrenal tumors, Conn’s adenoma, Adrenocortical hyperplasia, Cushing syndrome, Adrenal cancer, Pheochromocytoma

## Abstract

The last 10 years have produced an amazing number of significant discoveries in the field of adrenal endocrinology. The development of the adrenal gland was linked to specific molecules. Cortisol-producing lesions were associated mostly with defects of the cyclic AMP (cAMP) signaling pathway, whereas aldosterone-producing lesions were found to be the result of defects in aldosterone biosynthesis or the potassium channel *KCNJ5* and related molecules. Macronodular adrenal hyperplasia was linked to *ARMC5* defects and new genes were found to be involved in adrenocortical cancer (ACC). The succinate dehydrogenase (SDH) enzyme was proven to be the most important molecular pathway involved in pheochromocytomas, along with several other genes. Adrenomedullary tumors are now largely molecularly elucidated. Unfortunately, most of these important discoveries have yet to produce new therapeutic tools for our patients with adrenal diseases: ACC in its advanced stages remains largely an untreatable disorder and malignant pheochromocytomas are equally hard to treat. Thus, the challenge for the next 10 years is to translate the important discoveries of the previous decade into substantial advances in the treatment of adrenal disorders and tumors.

## Introduction

Adrenal endocrinology has always stood at the forefront of what is today called “precision medicine”: genetic disorders of adrenal steroidogenesis were among the first endocrine diseases to be molecularly elucidated in the 1980s. Neonatal screening for the various defects leading to congenital adrenal hyperplasia (CAH) in the 1990s was the successful outcome of the discoveries of the previous decade. In the last 10 years, a tremendous volume of new information has emerged from discoveries in the genetics of adrenocortical development, the biology of cortical and medullary tumors, and the elucidation of adrenal hormonogenesis (Table 1). This review does not cover all these discoveries, its emphasis being on adrenal endocrine oncology. Finally, instead of trying to cover all that is new in each disease, we attempted to provide information that can be used by the clinician in daily practice.

**Table 1:** Adrenal endocrinology advances 2007–2017

1. The importance of the cAMP-signaling pathway in benign cortisol-producing lesions	
2. Adrenocortical hypoplasia and development: a number of new genes	
*3. ARMC5* and the adrenal: a new gene for macronodular adrenal hyperplasia	
4. Genetics of food-dependent Cushing syndrome	
5. Aldosterone-producing lesions: *KCNJ5* and other genes	
6. Adrenocortical cancer: new genetics, but after mitotane, what?	
7. LC/MS: the new frontier in accurately detecting steroid production	
8. Subclinical syndromes: hypo/hyper-function and aldosterone and/or cortisol production	
9. Succinate dehydrogenase (SDH) mutations cause not only pheochromocytomas	
10. The other (non-SDH) genes in pheochromocytoma predisposition	

## The PKA pathway and benign cortisol-producing lesions

Cushing syndrome (CS) is caused by a primary adrenocortical process in 20–30% of cases, with cortisol-producing adrenal adenomas (ACA) responsible for 10–15% of cases and adrenocortical carcinomas (ACC) accounting for less than 5% of cases. In approximately 10% of adrenal CS, bilateral adrenal hyperplasia (BAH) is the cause. The most common forms of BAH are ACTH-independent macronodular adrenal hyperplasia (AIMAH), primary pigmented nodular adrenocortical disease (PPNAD), and isolated micronodular adrenocortical disease (iMAD). Adrenocortical nodules in AIMAH are greater than 1 cm in diameter, whereas in PPNAD and iMAD, nodules are less than 1 cm in diameter.

The cAMP-protein kinase A (cAMP-PKA) pathway plays a key role in the regulation of adrenocortical cell development, proliferation, and function. In the adrenocortical cell, this pathway is activated by the binding of ACTH to the ACTH receptor, a G protein-coupled receptor (GPCR) encoded by the *MC2R* gene. This leads to activation of adenylyl cyclase, synthesis of cAMP, and activation of protein kinase A (PKA). PKA is a heterotetramer comprised of two regulatory and two catalytic subunits.

Four isoforms exist for both the regulatory subunits (R1α, R1β, R2α, and R2β) and catalytic subunits (Cα, Cβ, Cγ, and PRKX) of PKA, with each isoform having individual localization and specificity [1]. Once cAMP binds to PKA, the catalytic subunits dissociate from the regulatory subunits, allowing the catalytic subunits to then phosphorylate downstream cytoplasmic and nuclear targets. Aberrantly increased cAMP-PKA signaling is implicated in the pathogenesis of most benign cortisol-producing tumors of the adrenal gland.

The study of rare genetic disorders with adrenal tumors has helped elucidate and highlight the central role played by the cAMP-PKA pathway in the development of CS. The first example of this was the discovery, 26 years ago, of the key role of cAMP-PKA signaling in the pathogenesis of cortisol-producing tumors in infants with McCune-Albright syndrome (MAS). MAS results from early embryonic postzygotic somatic activating mutations of the *GNAS* gene, which encodes the α-subunit of the stimulatory G protein (G_s_α) [2]. These mosaic gain-of-function mutations lead to constitutive activation of adenylyl cyclase, with such manifestations as café-au-lait skin macules, skeletal fibrous dysplasia, and multiple endocrinopathies, including precocious puberty, testicular and thyroid lesions, phosphate wasting, growth hormone excess, and, rarely, neonatal hypercortisolism [3, 4]. In MAS, hypercortisolism results from bilateral adrenocortical hyperplasia that develops from adrenocortical cells with fetal features and is termed primary bimorphic adrenocortical disease [5].

Another example of a rare genetic disorder that has provided insight into the crucial role of the cAMP-PKA pathway in CS is PPNAD in patients with Carney complex (CNC) [6]. CNC is a rare, autosomal dominant, multiple endocrine neoplasia syndrome manifesting with abnormal cutaneous and mucosal pigmentation, myxomatous tumors of the heart, skin and other tissues, psammomatous melanotic schwannomas, testicular tumors, breast ductal adenomas, osteochondromyxomas, and endocrine tumors with or without hormone overproduction. Endocrine tumors in this condition can include pituitary adenomas, PPNAD, thyroid tumors, and others [7]. Germline inactivating mutations in the *PRKAR1A* gene, situated at the 24.2–24.3 band of the long arm of chromosome 17, are found in more than 70% of patients with CNC and 80% of patients with CNC and PPNAD [8–10]. This gene encodes the regulatory subunit type 1α (R1α) of PKA, with CNC being the first known disease to be caused by mutations in the PKA enzyme. Inactivating mutations of *PRKAR1A* lead to constitutive activation of the cAMP-PKA pathway through loss of regulation of the catalytic subunits of PKA. Somatic mutations in *PRKAR1A* have also rarely been described in cortisol-producing adrenal tumors. In a study including 44 sporadic adrenocortical tumors (29 adenomas and 15 cancers), 17q22-24 losses were noted in 23% of adenomas and 53% of cancers, with inactivating *PRKAR1A* mutations identified in three tumors [11]. Mice lacking *PRKAR1A* specifically in the adrenal cortex develop autonomous excess cortisol secretion, dysregulated adrenocortical cell differentiation with increased proliferation and resistance to apoptosis, as well as improper maintenance and expansion of cortisol-producing fetal adrenocortical cells with regression of the adult adrenal cortex, leading to BAH and CS [12].

Mutations in cyclic nucleotide phosphodiesterases (PDEs) have also been implicated in the pathogenesis of adrenocortical tumors and CS. PDEs, which are the only known enzymes that degrade cyclic nucleotides, play a crucial role in the regulation of the cAMP-PKA pathway through regulating cAMP levels. In a single-nucleotide polymorphism-based, genome-wide association study of individuals with micronodular adrenocortical hyperplasia not caused by genetic defects in *GNAS* or *PRKAR1A*, mutations in genetic loci harboring PDE genes were most likely to be associated with the disease, with inactivating mutations of the *PDE11A* gene being the most commonly linked, followed by the *PDE8B* gene [13]. In addition, two missense substitutions that are relatively frequent polymorphisms of the *PDE11A* gene, R804H and R867G, were found with increased frequency among individuals with adrenal lesions [14]. Furthermore, in a study aimed at identifying the presence of germline or somatic *PDE11A* mutations that included 117 subjects with adrenocortical tumors and 195 control subjects, a higher frequency of *PDE11A* mutations was found in subjects with adrenocortical tumors compared to age/sex matched controls (16% vs 10% in adrenocortical cancer, 19% vs 10% in adrenocortical adenoma, and 24% vs 9% in AIMAH) [15]. In another series of 150 patients with CNC, a higher frequency of *PDE11A* variants was noted in patients with CNC compared with healthy controls [16]. Among patients with CNC, those with PPNAD and/or testicular large-cell calcifying Sertoli cell tumors (LCCSCT) were more frequently carriers of *PDE11A* variants compared to those without PPNAD and/or LCCSCT, respectively [16]. This suggests that PDE11A may be a genetic modifying factor for the development of adrenal and testicular tumors in patients with CNC and *PRKAR1A* mutations. *PDE11A* inactivating mutations may also play a role in the development of prostate cancer [17]. A single germline *PDE8B* missense substitution was initially reported in a patient with iMAD and CS [18]. The patient inherited the *PDE8B* mutation from her father, who was not diagnosed with CS but did have a phenotype consistent with mild iMAD with elevated midnight cortisol. In addition, in a case-control study of 216 unrelated patients with adrenocortical tumors and 192 controls, nine different *PDE8B* sequence changes were identified in the patients and controls, with two variations that were seen only in the patient group, demonstrating significant potential to impair protein function in vitro and in silico [19]. Furthermore, in a study of samples from 27 patients with adrenocortical tumors without mutations in *GNAS*, *PRKAR1A*, *PDE11A*, or *PDE8B*, abnormalities of the cAMP-signaling pathway were found, with mutation-negative ACAs having significantly decreased PDE activity [20].

The pathogenesis of isolated ACAs has also been linked to the cAMP-PKA pathway. A study performing whole exome sequencing of tumor-tissue specimens from 10 patients with unilateral ACAs and overt CS revealed somatic mutations in the *PRKACA* gene, encoding the catalytic subunit C*α* of PKA, in 8 out of 10 adenomas [21]. Further sequencing of an additional 129 adenomas revealed a Leu206Arg variant in 14 of these 129 ACAs, with all 14 patients having overt CS [21]. *PRKACA* mutations were found only in patients with overt CS and were associated with a more severe phenotype. Following the publication of these data in 2013, another three studies from China, Japan, and the USA demonstrated similar findings, with *PRKACA* mutations identified in 86 of 206 (42%) reported cases of ACAs with overt CS [22–26]. Furthermore, comparative genomic hybridization of samples from 35 patients with cortisol-secreting BAH and overt CS demonstrated germline copy-number gains (both inherited and de novo) in a region on chromosome 19p that includes the *PRKACA* gene in 5 patients [21]. The types of adrenocortical hyperplasia observed included PPNAD, iMAD, and AIMAH, with an increasing number of *PRKACA* gene copies correlating with a more severe phenotype [21, 26]. Activating mutations of *PRCACA* abolish the interaction between the regulatory and catalytic subunits of PKA, leading to constitutive activation of PKA.

AIMAH can be attributed to several different etiologies and accounts for < 1% of adrenal CS. Rarely, it is a component of multiple tumor syndromes, such as familial adenomatous polyposis (inactivating mutations in the *APC* gene), multiple endocrine neoplasia type 1 (MEN1 [loss of function mutations in the *MEN1* gene]), or hereditary leiomyomatosis and renal cell carcinoma (inactivating mutations in the *FH* gene) [27–29]. These were the only genetic defects linked to AIMAH until inactivating mutations of the *ARMC5* gene, which encodes Armadillo repeat containing protein 5, were found in 18 of 33 patients (55%) with AIMAH, with *ARMC5* likely acting as a tumor suppressor gene [30] (see below).

AIMAH has also been linked to ectopic or abnormal expression of hormone receptors linked to steroidogenesis in adrenocortical cells, primarily members of the GPCR family, including those for gastric inhibitory polypeptide (GIP), vasopressin (V1-vasopressin), β-adrenergic agonists, LH/hCG, or serotonin. These receptors were identified less frequently in adrenocortical adenomas and ACC. In a study of 20 patients with CS due to adrenocortical lesions, 6 out of 6 patients with AIMAH were observed to have abnormal receptor expression in adrenocortical cells with significant elevation in plasma cortisol after stimulation of these receptors [31]. This abnormal receptor expression may lead to potential therapeutic targets, as described in a 63-year-old woman that had AIMAH with abnormal LH/hCG and serotonin receptor expression, who responded to treatment with leuprolide [32]. AIMAH is a distinct and heterogeneous clinical and genetic entity. It is usually isolated or sporadic, with autosomal dominant inheritance when hereditary. Patients with AIMAH tend to present at an older age and often have an insidious clinical course with atypical CS over many years, but can occasionally develop severe CS. This was demonstrated in a study of 82 patients with adrenocortical tumors that compared 16 patients with AIMAH and subclinical cortisol secretion or atypical CS to patients with ACAs, aldosterone-producing adrenocortical adenomas (APA), and single non-functional adenomas [33]. In this study, patients with AIMAH demonstrated the highest urine 17-hydroxycorticosteroid excretion, even when urinary free cortisol was within or near the normal range. Furthermore, these patients also had high prevalence of abnormal or ectopic receptor expression. Molecular analysis of peripheral and/or tumor DNA in this cohort revealed germline mutations in the *APC* gene, *MEN1* gene, and *FH* gene in three of the patients with AIMAH, who did not have a family history of CS. Three other patients with AIMAH and a family history of CS had apparent autosomal dominant inheritance. A *PDE11A* variant was found in a patient with familial AIMAH, while a somatic *GNAS* mutation was found in the adrenal nodules of another patient. No mutations were identified in any of the above tested genes in the patients in the other groups. In addition, it was noted that patients with AIMAH from this cohort could be subgrouped into two histologic categories: type I AIMAH or bilateral macronodular adrenocortical hyperplasia (BMAH) and, more commonly, type II AIMAH. Adrenocortical tissue from patients with type I AIMAH demonstrated multiple nodules or discrete adenomas with intervening atrophic tissue, whereas adrenocortical tissue from patients with type II AIMAH showed diffuse hyperplasia with no residual normal or surrounding atrophic tissue. Most patients with AIMAH had histology consistent with type II AIMAH, whereas the three familial cases, the patients with germline *MEN1* and *APC* mutations, and the one patient with the somatic *GNAS* mutation demonstrated histology consistent with type I AIMAH. Another study of 14 patients with AIMAH revealed a link to aberrant cAMP-PKA pathway signaling through the demonstration of somatic losses of the 17q22-24 region in 73% of samples as well as PKA subunit and enzymatic activity changes [34]. Furthermore, the clinical and molecular heterogeneity in AIMAH was *shown* in a study of the expression profile of AIMAH through genomic cDNA microarray analysis of RNA extracted from eight tissues, including three tissues with GIP-dependent AIMAH. GIP-dependent AIMAH was found to be molecularly distinct from non-GIP-dependent AIMAH, with overexpression of the genes encoding the 7B2 protein (*SGNE1*) and WNT1-inducible signaling pathway protein 2 (*WISP2*) [35]. Lastly, another study analyzing integrated transcriptomic and genomic data from different nodules in the same patient with AIMAH showed that larger adrenal lesions in AIMAH accumulate an increased number of genomic and, subsequently, transcript abnormalities that are likely responsible for the progression from small nodules with mainly tissue metabolic derangements to large lesions with aberrant expression of oncogenic pathways [36].

## ARMC5 and the adrenal: a new gene and the disease that is inherited

A genetic origin of AIMAH was suggested by the bilateral nature of the disease, as well as cases of reported familial AIMAH and the description of AIMAH in patients with familial tumor syndromes. Investigation into the genetic origin of AIMAH led to the genotyping (both blood and tumor) of 33 patients with AIMAH, with detection of inactivating *ARMC5* gene mutations in 55% (18/33) of tumors, as noted above [30]. In all 18 cases of *ARMC5* mutations, both *ARMC5* alleles were mutated, one germline and one somatic, with four cases with germline mutations harboring nodule-specific secondary *ARMC5* mutations. This suggests the role of *ARMC5* as being a tumor-suppressor gene, with tumor development as described in Knudson’s two-hit hypothesis, when a secondary somatic inactivating mutation occurs in one allele in the setting of a preexisting germline inactivating mutation in the alternate allele. The function of *ARMC5* is unknown, though the protein it encodes contains an armadillo repeat domain, which is similar to the gene for β-catenin that also contains armadillo repeats and is often mutated in various cancers, including adrenocortical tumors [37]. In this study of 33 patients with AIMAH, inactivation of *ARMC5* was associated with reduced expression of steroidogenic enzymes and MC2R with abnormal cortisol production. In these patients, a gradual process of adrenocortical cell dedifferentiation and growth of bilateral masses was apparent. It is likely that hypercortisolemia in this disease is related more to increased adrenocortical mass than to cortisol overproduction per se. The large size of the adrenal glands may be related to loss of the ability to induce apoptosis in adrenocortical cells with *ARMC5* mutations, as demonstrated experimentally in human adrenocortical cell lines compared to wild-type cell lines [38]. In addition, an association between primary hyperaldosteronism and *ARMC5* mutations was first described in 2015 [39]. Lastly, though AIMAH was so named as it was deemed to be an ACTH-independent process, studies have shown that adrenal cortisol secretion in patients with AIMAH may be partially regulated through ACTH secretion from clusters of adrenocortical cells by paracrine action [40]. Thus, the more appropriate name for this disorder may be primary bilateral macronodular adrenal hyperplasia (PBMAH).

## Food-dependent Cushing syndrome: the new genetics

Food-dependent or GIP-dependent CS is associated with abnormal expression of the glucose-dependent insulinotropic polypeptide receptor (GIPR) in ACAs or AIMAH. GIPR is a GPCR. GIP-dependent CS was first described in 1992, in a 48-year-old woman with CS and BAH who had low-to-normal fasting plasma cortisol concentrations and elevated postprandial cortisol that was not suppressed by dexamethasone [41]. Her cortisol concentrations increased in response to test meals but not in response to intravenous glucose infusion, with increase in plasma cortisol paralleling the increase in GIP after meals. She also demonstrated increased plasma cortisol with intravenous GIP infusion, with subsequent adrenal tissue cell studies demonstrating increased cortisol production when adrenocortical cells were stimulated by GIP. Inappropriate postprandial stimulation of the GIPR in the adrenal by GIP leads to activation of the cAMP-PKA pathway, which results in adrenal cell proliferation and excess cortisol production. A study investigating the molecular pathogenesis of ectopic *GIPR* expression in adrenal tissue demonstrated that *GIPR* expression occurred through transcriptional activation of a single allele of the *GIPR* gene, with three of 15 tumor samples demonstrating somatic duplications in the chromosome region 19q13.32 containing the *GIPR* locus [42]. In two of these three samples, the duplicated 19q13.32 region was rearranged with other chromosome regions. These chromosome rearrangements did not result in gene fusion but rather placed the *GIPR* gene in a genomic environment near *cis*-acting regulatory regions favoring transcriptional activation. In the third sample, 19q duplication without chromosome rearrangement was identified.

## Aldosterone-producing lesions: *KCNJ5* and other genes

Physiologic aldosterone synthesis is stimulated by intravascular volume depletion and/or hyperkalemia. Intravascular volume depletion causes release of angiotensin II through the renin-angiotensin system, which binds to a GPCR on the zona glomerulosa cells. Furthermore, the resting membrane potential on these cells is set by potassium channel activity. These stimuli can cause membrane depolarization and activation of voltage-gated calcium channels, which leads to increased intracellular calcium, with resulting increase in aldosterone production and glomerulosa cell proliferation. Secreted aldosterone acts on the kidneys through increased sodium reabsorption and potassium excretion. Primary hyperaldosteronism (PA) is the most common form of secondary hypertension and is estimated to account for approximately 8% of cases of hypertension [43, 44]. The two most common causes of primary hyperaldosteronism are APAs (35%) and idiopathic hyperaldosteronism (65%), with less common causes being unilateral hyperplasia, aldosterone-producing adrenocortical carcinomas, and familial hyperaldosteronism (FH) [44]. Four forms of familial hyperaldosteronism (FH) have been described. These are inherited in an autosomal dominant fashion and make up 1–5% of cases of PA. The first form of FH is FH I or glucocorticoid remediable hyperaldosteronism (GRA). This was first described in a single family in 1966, with discovery of the causative chimeric gene 26 years later [45, 46]. GRA occurs as the result of the fusion of the promoter region of the *CYP11B1* gene, encoding 11β-hydroxylase (catalyzes the conversion of 11-deoxycortisol to cortisol), with *CYP11B2*, that encodes aldosterone synthase (converts deoxycorticosterone to corticosterone and 18-hydroxycorticosterone to aldosterone). This results in ACTH-dependent activation of aldosterone synthase and aldosterone overproduction. FH II does not have a known genetic cause, though mutations have been mapped to chromosome 7p22 [47, 48]. FH IV is attributed to germline mutations in *CACNA1H* (calcium channel, voltage-dependent, T-type, alpha-1H subunit), which encodes a T-type calcium channel [49]. Germline mutations in *KCNJ5* (potassium inwardly rectifying channel, subfamily J, member 5) lead to FH III, while somatic mutations are associated with 40% of APAs [50]. In a study of 22 patients with APAs, two recurrent somatic mutations (G151R and L168R), in and near the selectivity filter of the potassium channel KCNJ5, were present in eight of 22 APA samples. *KCNJ5* encodes an inwardly rectifying potassium channel, with the described mutations causing altered K+ channel selectivity. This leads to increased sodium conductance and cell depolarization, with resulting increased intracellular calcium and calcium signaling that leads to increased *CYP11B2* mRNA expression and aldosterone overproduction. When this process is chronic, it results in cell proliferation with clonal expansion of cells and eventual adenoma formation. These data from APAs were suggestive that inherited mutations in *KCNJ5* could lead to PA with BAH through a similar pathogenetic mechanism that affects all adrenocortical cells. This was seen in a family with PA and massive adrenal hyperplasia, who were heterozygous for the T158A mutation in the *KCNJ5* gene [51]. These implicate Germline heterozygous *KCNJ5* mutations have also been linked to the pathogenesis of hypertension associated with increased aldosterone response to ACTH stimulation. This was demonstrated in two hypertensive patients without PA that had radiologically “normal” adrenal glands and negative genetic testing for the *CYP11B1/CYP11B2* chimeric gene, who exhibited enhanced ACTH-dependent aldosterone secretion and response to treatment with a mineralocorticoid receptor antagonist [52]. In addition to the previously described *KCNJ5* and *CACNA1H* mutations, further molecular analysis has resulted in the elucidation of several other somatic and, less frequently, germline mutations associated with FH and/or sporadic APAs. These mutations include those in ATPase, Na^+^/K^+^ transporting, α1-polypeptide (*ATP1A1*), ATPase, Ca^2+^-transporting, plasma membrane, 3 (*ATP2B3*), and calcium channel, voltage-dependent, L-type, alpha-1D subunit (*CACNA1D*) [53–55]. Somatic mutations in these five genes have been identified in approximately 50% of APAs [54, 56]. Furthermore, a study investigating *CYP11B2* expression in tumor tissue from 51 patients with APAs suggested that tumorigenesis in APAs can occur within preexisting nodules through the acquisition of somatic mutations, possibly via a two-step process [57]. Of this patient cohort, seven patients (14%) demonstrated heterogeneity in *CYP11B2* expression. Of these seven patients, six had APAs with intratumor heterogeneity, with five tumors having distinct CYP11B2-positive and CYP11B2-negative tumor regions. Three of these six tumors had aldosterone-regulating mutations in *KCNJ5*, *ATP1A1*, or *CACNA1D* only in CYP11B2-positive regions, while one had two different mutations in *KCNJ5* and *ATP2B3* in two histologically different CYP11B2-positive regions. The seventh patient had a multinodular hyperplasic adrenal that showed CYP11B2-positive and CYP11B2-negative nodules, and different mutations in each CYP11B2-positive nodule (*ATP1A1*, *CACNA1D*), with no mutations in CYP11B2-negative nodules or the adjacent normal adrenal.

## Adrenocortical cancer: new genetics, but after mitotane, what?

Adrenocortical carcinoma (ACC) is a rare and aggressive disease. Most cases are sporadic, though ACCs have been described in at least eight hereditary cancer syndromes, including Li-Fraumeni syndrome (*TP53* gene), Beckwith-Wiedemann syndrome (abnormalities in 11p15), MEN1 (*MEN1* gene), familial adenomatous polyposis (*FAP* gene), neurofibromatosis type 1 (*NF1* gene), and CNC (*PRKAR1A* gene) [58, 59]. Tumorigenesis in sporadic ACC has been hypothesized to be a multistep process. An example suggesting multistep tumor progression from a benign to malignant lesion is that of a 43-year-old patient who underwent unilateral adrenalectomy for an incidental adrenal mass, where pathology demonstrated two different tumor components. The central tumor component harbored ACC, but the surrounding (peripheral) tumor component was consistent with benign tumor. Molecular analysis of the tumor demonstrated 17p13 loss of heterozygosity (LOH), 11p15 uniparental disomy (UPD), and IGF-II gene overexpression, as well as chromosomal gains and losses in the malignant tissue, whereas the benign tissue did not demonstrate any abnormalities [60]. A study published in 2014 based on the molecular analysis of 45 ACCs further elucidated the molecular pathogenesis of ACC with reidentification of genetic alterations in known driver genes *CTNNB1*, *TP53*, *CDKN2A*, and *RB1*, as well as identification of driver genes not previously reported including, *ZNRF3*, *DAXX*, *TERT*, and *MED12* [61]. These new genes were validated by molecular analysis in an independent cohort of 77 ACCs. The most frequent genetic abnormality identified in 21% of tumors was that of the *ZNRF3* gene, a potential new tumor suppressor gene related to the β-catenin pathway which encodes a cell surface E3 ubiquitin ligase. Thirty-nine percent of tumors harbored an alteration affecting the Wnt/β-catenin pathway, while 33% harbored alterations affecting the p53-Rb pathway. Another study published in 2016, based on the comprehensive genomic characterization of 91 ACCs, expanded the list of known ACC driver genes to include *PRKAR1A*, *RPL22*, *TERF2*, *CCNE1*, and *NF1* [62]. In this study, somatic mutations were noted most frequently in the following genes: *TP53* (21%), *ZNRF3* (19%), *CDKN2A* (15%), *CTNNB1* (16%), *TERT* (14%), and *PRKAR1A* (11%). Furthermore, genome-wide DNA copy number analysis demonstrated increased frequency of DNA loss, followed by whole genome doubling, which was associated with more aggressive tumors.

ACC has a poor prognosis with 5-year survival in early-stage disease approaching 45–60%, and 5-year survival in advanced disease approaching 10–25%. Surgical resection is the only form of curative treatment and is the treatment of choice for non-metastatic ACC. Adjuvant therapies are employed to help reduce the risk of recurrence. Treatment of unresectable or metastatic ACC is considered palliative. In surgically resectable disease, adjuvant mitotane is used in patients with high-risk features, such as high-grade or incompletely resected disease, tumor spillage or fracture during resection, and/or tumors with vascular or capsular invasion. A cisplatin-based adjuvant chemotherapy regimen in addition to mitotane may be used for patients at high risk for early recurrence. Post-operative radiation may be considered in patients with high risk or stage III disease. For patients with inoperable, recurrent, or advanced disease, surgery can be considered for select patients, otherwise mitotane is an option for low-grade, slow-growing disease. The majority of patients require treatment with a combination of mitotane with etoposide, doxorubicin, and cisplatin (EDP) rather than streptozocin. A study from 2016 compared the combination of EDP plus mitotane versus streptozocin plus mitotane in the treatment of 304 patients with advanced ACC [63]. Patients receiving first-line therapy with EDP-mitotane had a significantly higher response rate and longer median progression-free survival compared to the streptozocin-mitotane group (5 months vs 2.1 months, respectively). However, overall survival was similar between the two groups.

## Succinate dehydrogenase (SDH) mutations: not only pheochromocytomas—the other (non-SDH) genes in pheochromocytoma/paraganglioma

Catecholamine-producing tumors of neural crest origin include pheochromocytomas that arise from the adrenal medulla, and paragangliomas that arise from extra-adrenal chromaffin cells of the thoracic, abdominal, and pelvic sympathetic paravertebral ganglia. Paragangliomas can also occur in the parasympathetic ganglia of the head and neck, located along the glossopharyngeal and vagal nerves. Pheochromocytomas occur in 80–90% of cases, whereas paragangliomas occur in 10–20% of cases. More than 30% of these tumors are familial. Fifteen different pheochromocytoma/paraganglioma susceptibility genes have been reported. These genes can be separated into two categories: those encoding proteins that function in the cellular response to hypoxia, found primarily in noradrenergic paragangliomas (*SDHA*, *SDHB*, *SDHC*, *SDHD*, *SDHAF2*, *VHL*, *HIF2*, *FH*, *EGLN1*, *EGLN2*, and *KIF1B* genes), and those that encode protein-activating kinases and are found primarily in adrenergic adrenal pheochromocytomas (*RET*, *NF1*, *MAX*, and *TMEM127*) [64–68]. *VHL*, *RET*, *NF1*, *SDHB*, and *SDHD* are major susceptibility genes, accounting for 90% of familial pheochromocytomas/paragangliomas, whereas *TMEM127*, *SDHA*, *SDHC*, *SDHAF2*, and *MAX* are minor susceptibility genes responsible for 10% of these tumors. Features that should raise suspicion for a hereditary cause of pheochromocytoma/paraganglioma include family history, syndromic features, and multifocal, bilateral, and/or metastatic disease. Six autosomal dominant pheochromocytoma/paraganglioma syndromes have distinct clinical features and include neurofibromatosis type 1 (NF1), multiple endocrine neoplasia type 2 (MEN2), von Hippel-Lindau syndrome (VHL), renal cell carcinoma with *SDHB* mutation, Carney triad, and Carney-Stratakis syndrome (CSS). The O-methylated metabolites of catecholamines, plasma metanephrine (epinephrine metabolite), normetanephrine (norepinephrine metabolite), and methoxytyramine (dopamine metabolite) can help distinguish the different familial forms of pheochromocytoma and paraganglioma linked to the major susceptibility genes [69]. In a study of 173 patients with pheochromocytoma and/or paraganglioma, all patients with *MEN2* and *NF1* mutations had elevated plasma metanephrine, whereas patients with *VHL* mutations usually had elevated normetanephrine, and 70% of patients with *SDHB* and *SDHD* mutations had elevated plasma methoxytyramine. Measurement of normetanephrine and metanephrine allowed for the distinction of those patients with *MEN2* and *NF1* from those with *VHL*, *SDHB*, and *SDHD* mutations in 99% of cases, whereas plasma methoxytyramine measurement allowed for discrimination of *VHL* mutations from *SDHB* and *SDHD* mutations in 78% of patients. Measurement of metanephrines is more reliable than measurement of catecholamines (epinephrine, norepinephrine, and dopamine) for the diagnosis of pheochromocytoma/paraganglioma, as metanephrines are produced continuously and independently of catecholamine secretion, whereas catecholamines have an episodic profile.

The most frequent germline mutations in pheochromocytoma and paraganglioma are in the *SDHB* (10.3%) and *SDHD* (8.9%) genes [70]. The succinate dehydrogenase (SDH) subunit genes comprise mitochondrial complex II (*SDHB*, *SDHC*, *SDHD*, and *SDHA*). *SDHAF2* encodes SDH assembly factor 2. These genes act as tumor suppressor genes. SDH (or succinate-ubiquinone reductase) plays a role in the electron transport chain and tricarboxylic-acid (TCA) cycle. The SDH enzyme complex is located in the mitochondrial matrix and has four subunits. SDHA and SDHB comprise the catalytic subunits of SDH. These catalytic subunits are anchored to the mitochondrial inner membrane by SDHC and SCHD, which harbor the ubiquinone binding site. The SDH enzyme couples the oxidation of succinate to fumarate in the TCA cycle, with the electron transfer to the terminal acceptor ubiquinone in the electron transport chain in a manner that prevents the formation of reactive oxygen species [71].

*SDHB* mutations primarily predispose to extra-adrenal paragangliomas that secrete dopamine and norepinephrine and tend to occur at a young age, with high malignant potential and an aggressive disease course. These mutations less commonly lead to adrenal pheochromocytomas or head and neck paragangliomas. *SDHB* mutations are found in 40% of patients with metastatic pheochromocytoma/paraganglioma. *SDHD* gene mutations are associated with multifocal head and neck paragangliomas and less commonly with adrenal pheochromocytomas or paragangliomas at other sites. These mutations are inherited paternally, likely in the setting of maternal imprinting. *SDHD*-associated paragangliomas are rarely malignant, though 20–30% may be biochemically active and may secrete methoxytyramine. Of 263 patients with pheochromocytoma/paraganglioma evaluated at our institution from 2000 to 2010, 125 patients were found to have metastatic disease with 32 of these patients presenting before the age of 20. Of these 32 patients, 71.8% had primary extra-adrenal tumors with genetic analysis demonstrating germline *SDHB* mutations in 71.9% of patients, *SDHD* mutations in 9.4% of patients (found in all patients with head and neck paraganglioma), and *VHL* mutations in 6.3% of patients [72]. The average age at diagnosis of pheochromocytoma/paraganglioma in this pediatric group was 13 years (range 5 to 19 years). All five deceased pediatric patients harbored the *SDHB* mutation. Given these data, it was recommended that patients with metastatic pheochromocytoma/paraganglioma presenting in childhood or adolescence undergo initial genetic testing for *SDHB* mutations, with the exception of patients with primary tumors of the head and neck (*SDHD* genetic testing recommended) and patients with a family history that suggests a different mutation. Screening for SDHB and SDHD mutations may be also be prudent in patients with paragangliomas in certain uncommon locations such as the mediastinum or the organ of Zuckerkandl. Mutations in *SDHB* or *SDHD* were identified in 10 out of 10 patients with mediastinal paragangliomas, a rare location for paraganglioma development (2%) [73]. Furthermore, *SDHB* or *SDHD* mutations were detected in the majority of patients with organ of Zuckerkandl paragangliomas, another rare site of paraganglioma occurrence located around the origin of the inferior mesenteric artery and extending to the level of the aortic bifurcation [74]. In addition, per the most recent *Endocrine Society Clinical Practice Guideline* published in 2014, patients with paragangliomas should undergo genetic testing for SDH mutations, and those with metastatic disease should undergo evaluation for *SDHB* mutations [70]. Functional imaging may be useful in localization of these tumors. A study comparing the sensitivity of functional imaging techniques in the localization of head and neck paragangliomas (harboring *SDHD* or *SDHB* mutations) demonstrated that ^18^F-fluorodihydroxyphenylalanine (^18^F-FDOPA) positron emission tomography (PET) was the most sensitive study, localizing all tumors, followed by ^18^F-fluoro-2-deoxy-D-glucose (^18^F-FDG) PET/CT, which localized 77% of tumors^75^. ^111^In-pentetreotide scintigraphy, ^18^F-fluorodopamine (^18^F-FDA) PET/CT, and ^123^I-metaiodobenzylguanidine (^123^I-MIBG) scintigraphy had lower sensitivity, with ^123^I-MIBG scintigraphy localizing only 31% of tumors [75]. Another study, investigating the sensitivity of functional imaging in detecting metastases in patients with *SDHB*-associated paraganglioma demonstrated an ^18^F-FDG PET/CT sensitivity approaching 100% [76]. ^18^F-FDA PET and ^123^I-MIBG scintigraphy failed to detect metastases in 30% and 35% of body regions, respectively, with ^18^F-FDG PET/CT detecting at least 90% of metastases in these regions. ^18^F-FDA PET and ^123^I-MIBG scintigraphy may be less sensitive in detecting metastatic paraganglioma due to tumor dedifferentiation, whereas metastatic tumors have an increased metabolic rate, which makes them more likely to be detected by^18^F-FDG PET/CT compared to primary tumors. The two previous studies suggest that ^18^F-FDOPA PET may be the imaging modality of choice for head and neck paraganglioma, whereas ^18^F-FDG PET/CT should be the imaging modality of choice for detecting metastatic paraganglioma. Furthermore, false-negative ^123^I-MIBG scintigraphy in patients with pheochromocytoma or paraganglioma has been associated with a more aggressive disease course and the presence of *SDHB* mutation [77].

SDH deficiency is also implicated in two aforementioned pheochromocytoma/paraganglioma syndromes, Carney triad and CSS. Carney triad, or the association of gastric leiomyosarcoma or gastrointestinal stromal tumor (GIST), functional extra-adrenal paraganglioma, and pulmonary chondroma, was first described in 1977 [78]. Carney triad is a rare, non-hereditary syndrome with a chronic, indolent disease course that predominantly affects women, with onset primarily before 30 years of age [79]. Adrenocortical tumors are a fourth component of Carney triad. In 2007, evaluation for a genetic cause of Carney triad through comparative genomic hybridization of DNA samples from 41 patients, with the aim of detecting common gene abnormalities associated with paragangliomas (inactivating mutations in *SDHA*, *SDHB*, *SDHC*, and *SDHD*, or collectively *SDHx*) and GISTs (activating mutations in *KIT* and *PDGFRA*), did not identify abnormalities in these genes [80]. However, this evaluation did identify abnormalities in chromosome 1, most frequently deletion of the 1q12-q23.3 chromosomal region harboring the *SDHC* gene, as well as loss of the 1p region. In 2009, immunohistochemical analysis of tumor samples from patients with Carney triad demonstrated a loss of the SDHB protein [81]. Finally, in 2014, evaluation of the DNA methylation patterns of all four SDH subunits demonstrated abnormal DNA hypermethylation of the *SDHC* gene in all tumors of patients with Carney triad, with reduced *SDHC* mRNA expression [82]. Subsequently, germline mutations have rarely been described in patients with Carney triad [83]. This was demonstrated in a study involving the largest cohort of Carney triad patients available internationally that demonstrated germline variants of *SDHA*, *SDHB*, or *SHDC* in six out of 63 patients (9.5%). Another syndrome, CSS, was first described in 2002 and is characterized by the dyad of GISTs and paragangliomas [84]. It is an autosomal-dominant disorder with incomplete penetrance that affects both genders beginning in childhood and adolescence. Patients with CSS harbor inactivating germline mutations in the *SDHx* genes [85]*.*

*SDH* mutations have also been associated with wild-type (WT) GISTs [86]. GISTs are the most common mesenchymal tumors of the gastrointestinal tract. These tumors are categorized into two groups according to their pathogenic genetic abnormality. The larger group is comprised of GISTs that harbor mutations in *KIT* (75–80% of tumors) and *PDGFRA* (5–15% of tumors) [87–89]. The second group is comprised of the remaining 10% of GISTs and is further divided into SDH-deficient (*SDHx* abnormalities) and non-SDH-deficient tumors (mutations in *NF1*, *BRAF*, *KRAS*, *PIK3CA*, and the *ETV6-NTRK3* fusion gene) [90–97]. In a study of 34 patients with sporadic WT GISTs without a family history of paraganglioma, 12% of patients were found to have germline mutations in *SDHB* or *SDHC*, while those who did not harbor a detectable *SDH* mutation demonstrated significantly decreased SDHB protein expression. In another study of 95 tumor specimens from pediatric patients with WT GISTs, 84 specimens were classified as SDH-deficient. Of these 84 tumors, 67% had *SDH*x mutations, with 82% of these mutations representing germline mutations. Twenty-two percent of the SDH-deficient tumors had hypermethylation of the *SDHC* promoter. Findings from these two studies highlight the possible central role of SDH dysregulation in the development of WT GISTs [97].

Pituitary adenomas have rarely been described in patients with pheochromocytomas/paragangliomas, but have been frequently observed in patients with MEN1 [98–101]. MEN1 is an autosomal dominant hereditary disorder due to mutations in the *MEN1* gene that lead to the development of tumors in the parathyroid glands, pancreas, and anterior pituitary. The first case of a patient who developed both paragangliomas and a growth hormone–secreting pituitary adenoma was described in 2012 [100]. This patient harbored a germline *SDHD* mutation that was identified in the pituitary tumor tissue. Evaluation of 39 cases of sporadic or familial pheochromocytoma/paraganglioma and pituitary adenomas for abnormalities in known pheochromocytoma/paraganglioma susceptibility genes (*SDHx*, *SDHAF2*, *RET*, *VHL*, *TMEM127*, *MAX*, and *FH*) and known pituitary adenoma genes (*MEN1*, *AIP*, and *CDKN1B)* demonstrated 11 germline mutations (*SDHB*, *SDHC*, and *SDHAF2*) and four variants of unknown significance (*SDHA*, *SDHB*, and *SDHAF2*) [101]. Analysis of tumor tissue from the pituitary adenomas and the pheochromocytomas/paragangliomas demonstrated *SDHB* LOH in three pituitary adenomas and LOH of *MEN1* in two pheochromocytomas. These findings suggest a common pathogenic mechanism in these cases. The possible role of *SDHx* mutations in the development of pituitary abnormalities was also demonstrated in a mouse model, where the pituitaries of *Sdhb*^+/−^ mice older than 12 months were found to have an increased number mainly of prolactin-secreting cells and several ultrastructural abnormalities, such as intranuclear inclusions, altered chromatin nuclear pattern, and abnormal mitochondria [102]. Mutant mice tended to have higher insulin-like growth factor-1 levels at all ages, whereas prolactin and growth hormone levels varied according to age and sex.

*SDHx* mutations have also been linked to other non-paraganglionic tumors, including pancreatic neuroendocrine tumors, abdominal ganglioneuromas, and renal cell carcinomas [103, 104].

## Adrenal endocrinology: the next 10 years

It is very clear that tremendous advances have been made in our understanding of what makes the adrenal gland function and what causes its diseases. But, as mentioned in the introduction and is evident from the items reviewed here, these discoveries have yet to be translated into new therapies. Diseases like adrenal cancer and malignant pheochromocytoma have no cure beyond complete surgical resection in their early stages, which is the way they were being treated decades ago. Thus, it is imperative that the next 10 years lead to translation of the molecular discoveries into new therapeutic options for our patients. Table 2 lists some of the anticipated directions for adrenal endocrinology in the next 10 years and it would be useful for the reader to contrast it with Table 1.

**Table 2:** Adrenal endocrinology in the next 10 years

1. Precision medicine in the adrenal: translate genetics into predictive medicine and new therapies	
2. Knowledge in adrenal development may lead to the use of organoids and adrenal regrowth/replacement in patients with adrenal failure	
3. Development of new treatments for adrenal cancer is desperately needed	
4. Malignant pheochromocytoma/paraganglioma also needs new treatment options	
5. There is a need for better medical treatment of adrenal insufficiency and subclinical steroid hypersecretion, both excess aldosterone and/or cortisol.	
